# The importance of hemolysis detection among neonates for interpretation of potassium results

**DOI:** 10.1093/labmed/lmae094

**Published:** 2024-12-03

**Authors:** Alan H B Wu

**Affiliations:** Department of Laboratory Medicine, University of California, San Francisco, CA, US

**Keywords:** pseudohyperkalemia, pseudoeukalemia, hemolysis, potassium analysis

## Abstract

Specimen hemolysis is a frequent finding when blood is collected from neonates. This produces artificially high results for some analytes, such as potassium. Testing samples for electrolytes using point-of-care (POC) blood gas analyzers is convenient and facile. However, unlike testing that is conducted on serum or plasma from a central laboratory, detection of hemolysis using POC analyzers cannot currently be achieved. As described in these cases, the presence of hemolysis can produce ambiguities and delays in the diagnosis and management of neonates.

## Introduction

Specimen hemolysis is a well-recognized source of preanalytical laboratory error. The lysis of cellular elements from blood, for example, red cells, white cells, and platelets, can release analytes found in high intracellular concentrations relative to serum to produce artificial increases in potassium, lactate dehydrogenase, aspartate aminotransferase, among other analytes. The reference range for potassium is narrow, typically from 3.5 to 5.0 mmol/L for adults and higher, at 4.0 to 6.0 mmol/L, for neonates.^1^ Any degree of hemolysis can produce a result that moves a test result from low to normal and from normal to abnormal. Mansour et al^2^ reported that potassium can increase from 0.40 to 0.51 mmol/L potassium for every 0.1 g/dL of cell-free hemoglobin. Neonates and infants are particularly vulnerable for the collection of hemolytic specimens because blood collection from this population, via heel sticks into capillary tubes, is particularly difficult due to the child’s weight, venous access, and limited total blood volume.^3^ Moreover, neonate red blood cells are more rigid, leading to fragility and shorter lifespan in the circulation.^4^ The amount of blood available from neonates, especially premature infants, is barely sufficient to conduct blood tests, and priority of testing is sometimes necessary.

Although electrolyte testing for adults and older children on serum or plasma is usually sent to the central laboratory, testing for electrolytes is available on whole blood samples using bedside using point-of-care (POC) blood gas analyzers. This approach produces results faster and requires less sample, as specimens are not centrifuged. Neonates have higher hematocrit values, that is, 42%-65%^5^; therefore, even more blood is needed to obtain the volume of serum needed for testing. Mahieu et al^6^ reported that after implementation of POC testing, there was a 48% reduction in transfusions per low-birth-weight infants caused by iatrogenic anemia relative to central laboratory testing.

The major disadvantage of whole blood electrolyte analysis is that specimens cannot be tested for the presence of hemolysis. The red color of hemoglobin is routinely measured using spectrophotometric methods by current central laboratory analyzers when serum or plasma is tested. Unfortunately, until just recently, hemolysis detection has not been available on whole blood samples due to the spectral absorbance of intact red cells.

I previously reported on the rate of hemolysis as detected in serum for potassium testing on adults conducted by a central laboratory.^7^ If the potassium result is abnormally high or low, the clinical laboratory alerts the medical team of an abnormality via a flag noted on the patient’s electronic medical record. A note of hemolysis should prompt further investigation, such as a request for a repeat blood collection. If the potassium result is normal, there is no abnormal flag produced. Therefore, even if there is a note for the presence of hemolysis on the laboratory report, the care team may not notice or understand that the actual potassium result may be low, and no action is taken. In my prior report, I particularly focused on the medical consequences of hemolysis for potassium results that were in the lower limit of the reference range (pseudoeukalemia).^7^ In these case reports, the consequences of undetected hemolysis for neonates is presented for both pseudoeukalemia and pseudohyperkalemia.

All infants in this study were admitted to our hospital’s neonatal intensive care unit. All potassium testing was conducted from the central laboratory.

## Clinical Histories

### Case 1: Pseudohyperkalemia

This patient was female and weighed 3570 g, born to a 22-year-old mother (gestation 35 weeks 6 days, late preterm infant, G1P0101) via a normal spontaneous vaginal. The infant had hypoglycemia (glucose 28 mg/dL), required continuous positive airway pressure (CPAP) for respiratory distress, and underwent sepsis evaluation. Serum potassium results on the first few days of life are shown in TABLE 1. All but 1 of the samples were hemolyzed.

**TABLE 1.** Potassium Results in Neonatal Blood Exhibiting Hemolysis

Hours of life	Potassium^a^	Alert
Patient 1		
32	5.4	Hemolysis
44	6.8 H	Hemolysis
72	4.4	None
92	6.3 H	Hemolysis
118	7.4 H	Hemolysis
144	6.4 H	Hemolysis
Patient 2		
28	5.5	None
40	4.0	None
52	3.5 L	Hemolysis

^a^Reference range: 4.0-6.0 mmol/L.

### Case 2: Pseudoeukalemia

This patient was a 33 cm long, 882 g female infant was delivered from a 33-year-old mother (gestation 41 weeks 7 days, G2P0111) via a low traverse caesarian section. The prenatal course was complicated by intrauterine growth restriction with signs of placental insufficiency. She had some respiratory distress and was treated with CPAP. She was given total parental nutrition with tropic feeds due to her intrauterine growth restriction. On day 2, there was a concern for a necrotizing enterocolitis, and the baby missed 3 feedings. There was also some emesis overnight. As a result, there was a suspicion of hypovolemia. TABLE 1 shows the serum potassium results.

## Discussion

In case 1, the potassium result on the first sample was within the reference range on the sample that was hemolyzed. As such, it is unknown whether this neonate was actually hypokalemic. The subsequent samples, taken 12 hours later, showed an increased potassium with hemolysis. It is unknown whether this patient had real or pseudo-hyperkalemia. The third sample, taken 26 hours later, was eukalemic with no hemolysis, suggesting that all of the previous samples had normal potassium results. The subsequent 3 samples were all hyperkalemic and hemolytic. It is unclear whether the neonate had a true hyperkalemia, for example, due to intravascular hemolysis, or if this was artificially increased. Repeat blood collection and potassium testing after review of these reports was not conducted because of concern for excess blood removal. It was possible that a repeat blood collection would still produce a hemolyzed sample.

In case 2, the first 2 values taken were within the normal range (4.0-6.0 mmol/L). The third value was hemolyzed, suggesting that this result was likely to be hypokalemic. A progress note made 4 hours after this third sample suggested the possibility of hypovolemia, a known cause of hypokalemia.^8^ A subsequent nonhemolyzed sample taken 12 hours later confirmed this deficiency.

## Conclusion

In each of these cases, the detection of hemolysis by the central laboratory provided neonatologists and nurses some guidance in the interpretation of potassium results. Had these samples been tested on a POC whole blood analyzer, no information on hemolysis status would have been produced, resulting in inappropriate interpretation of the potassium results. This can lead to inaccurate therapeutic decisions as well as delays in neonate discharge. There are now commercial blood gas analyzers that can detect hemolysis when whole blood testing is conducted.^9^ It is hoped that widespread adoption of this and similar technologies will lead to recommendations of improved laboratory practices by laboratory accreditation agencies such as the Centers for Medicare and Medicaid and the College of American Pathologists, as well as hospital accreditation agencies such as the Joint Commission.

## Abbreviations

POC: point-of-care
CPAP: continuous positive airway pressure
